# Supplementary material for: Development of chemokine network inhibitors using combinatorial saturation mutagenesis
Source: Commun Biol. 2025 Apr 3;8:549. doi: 10.1038/s42003-025-07778-6 (PMC11969024; doi:10.1038/s42003-025-07778-6)
Supplement: Supplementary file 4 — Supplementary Data 2 [file 42003_2025_7778_MOESM4_ESM.zip › SupplementaryData3/scripts/xAF3.tar!/./script/analysis/JK/plots/ckckstericplot.pdf]

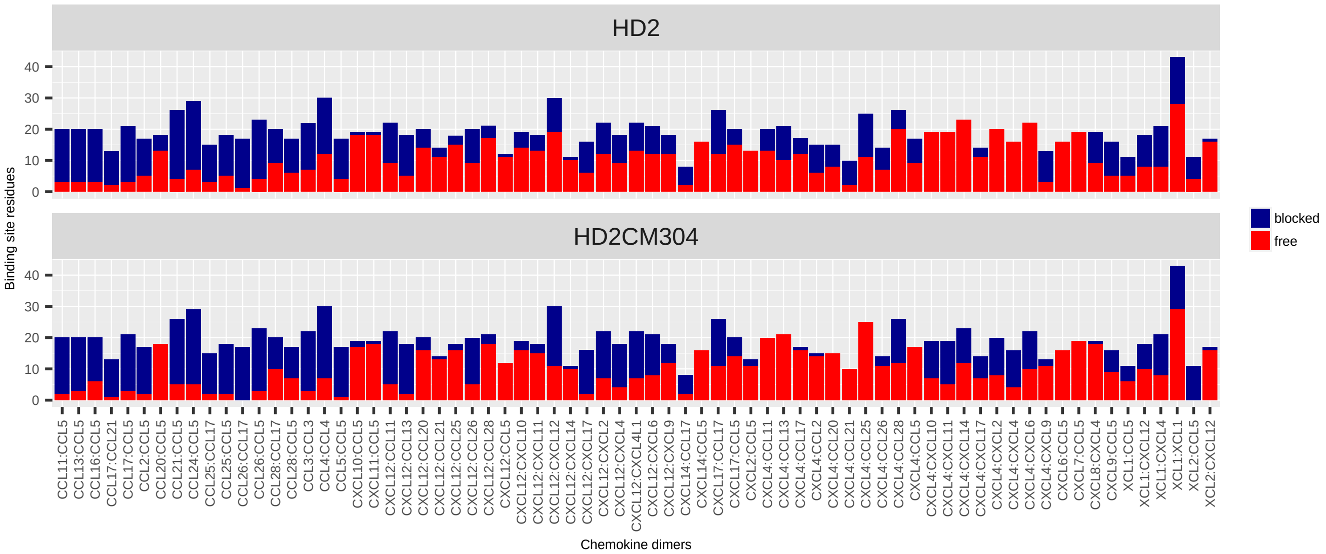
HD2
HD2CM304
Binding site residues
Chemokine dimers
blocked
free
0
10
20
30
40
CCL11:CCL5
CCL13:CCL5
CCL16:CCL5
CCL17:CCL21
CCL17:CCL5
CCL2:CCL5
CCL20:CCL5
CCL21:CCL5
CCL24:CCL5
CCL25:CCL17
CCL25:CCL5
CCL26:CCL17
CCL26:CCL5
CCL28:CCL17
CCL28:CCL5
CCL3:CCL3
CCL4:CCL4
CCL5:CCL5
CXCL10:CCL5
CXCL11:CCL5
CXCL12:CCL11
CXCL12:CCL13
CXCL12:CCL20
CXCL12:CCL21
CXCL12:CCL25
CXCL12:CCL26
CXCL12:CCL28
CXCL12:CCL5
CXCL12:CXCL10
CXCL12:CXCL11
CXCL12:CXCL12
CXCL12:CXCL14
CXCL12:CXCL17
CXCL12:CXCL2
CXCL12:CXCL4
CXCL12:CXCL4L1
CXCL12:CXCL6
CXCL12:CXCL9
CXCL14:CCL17
CXCL14:CCL5
CXCL17:CCL17
CXCL17:CCL5
CXCL2:CCL5
CXCL4:CCL11
CXCL4:CCL13
CXCL4:CCL17
CXCL4:CCL2
CXCL4:CCL20
CXCL4:CCL21
CXCL4:CCL25
CXCL4:CCL26
CXCL4:CCL28
CXCL4:CCL5
CXCL4:CXCL10
CXCL4:CXCL11
CXCL4:CXCL14
CXCL4:CXCL17
CXCL4:CXCL2
CXCL4:CXCL4
CXCL4:CXCL6
CXCL4:CXCL9
CXCL6:CCL5
CXCL7:CCL5
CXCL8:CXCL4
CXCL9:CCL5
XCL1:CCL5
XCL1:CXCL12
XCL1:CXCL4
XCL1:XCL1
XCL2:CCL5
XCL2:CXCL12
